# CXCL12 loaded-dermal filler captures CXCR4 expressing melanoma circulating tumor cells

**DOI:** 10.1038/s41419-019-1796-6

**Published:** 2019-07-22

**Authors:** Caterina Ieranò, Crescenzo D’Alterio, Simona Giarra, Maria Napolitano, Giuseppina Rea, Luigi Portella, Assunta Santagata, Anna Maria Trotta, Antonio Barbieri, Virginia Campani, Antonio Luciano, Claudio Arra, Anna Maria Anniciello, Gerardo Botti, Laura Mayol, Giuseppe De Rosa, Roberto Pacelli, Stefania Scala

**Affiliations:** 1 Functional Genomics, Istituto Nazionale Tumori – IRCCS - Fondazione “G. Pascale”, Napoli, Italy; 2 Department of Pharmacy, Federico II University, Napoli, Italy; 3 Animal Facility, Istituto Nazionale Tumori – IRCCS - Fondazione “G. Pascale”, Napoli, Italy; 4 Pathology Unit, Istituto Nazionale Tumori – IRCCS - Fondazione “G. Pascale”, Napoli, Italy; 5 Department of Advanced Biomedical Sciences, Federico II University School of Medicine, Napoli, Italy

**Keywords:** Metastasis, Melanoma

## Abstract

Development of distant metastasis relies on interactions between cancer and stromal cells. CXCL12, also known as stromal-derived factor 1α (SDF-1α), is a major chemokine constitutively secreted in bone marrow, lymph nodes, liver and lung, playing a critical role in the migration and seeding of neoplastic cells. CXCL12 activates the CXCR4 receptor that is overexpressed in several human cancer cells. Recent evidence reveals that tumors induce pre-metastatic niches in target organ producing tumor-derived factors. Pre-metastatic niches represent a tumor growth-favoring microenvironment in absence of cancer cells. A commercially available dermal filler, hyaluronic acid (HA) -based gel, loaded with CXCL12 (CLG) reproduced a “fake” pre-metastatic niche. In vitro, B16-hCXCR4-GFP, human cxcr4 expressing murine melanoma cells efficiently migrated toward CLG. In vivo, CLGs and empty gels (EGs) were subcutaneously injected into C57BL/6 mice and 5 days later B16-hCXCR4-GFP cells were intravenously inoculated. CLGs were able to recruit a significantly higher number of B16-hCXCR4-GFP cells as compared to EGs, with reduced lung metastasis in mice carrying CLG. CLG were infiltrated by higher number of CD45-positive leukocytes, mainly neutrophils CD11b+Ly6G+ cells, myeloid CD11b+Ly6G- and macrophages F4/80. CLG recovered cells recapitulated the features of B16-hCXCR4-GFP (epithelial, melanin rich, MELAN A/ S100/ c-Kit/CXCR4 pos; α-SMA neg). Thus a HA-based dermal filler loaded with CXCL12 can attract and trap CXCR4+tumor cells. The CLG trapped cells can be recovered and biologically characterized. As a corollary, a reduction in CXCR4 dependent lung metastasis was detected.

## Introduction

Cancer metastases contribute to over 90% of cancer deaths. The metastatic process is a complex process that involves primary tumors, tumor microenvironment and distant organs^1,2^. Recent studies suggest that stromal cells control the effective organ colonization and growth of circulating tumor cells (CTCs)^1^. CXCL12, also known as stromal-derived factor 1α (SDF-1α), is a major chemokine, constitutively secreted in bone marrow, lymph nodes, liver and lung^3^; it plays a key role in physiologic processes, such as lymphocyte homing, chemotactic migration,^4,5^ and metastatic development^6^. CXCL12 binds the chemokine receptor, CXCR4, overexpressed in at least 23 different human cancers^7–9^ and CXCR7, a recently identified CXCL12 receptor also overexpressed in tumors^10–12^ . Fibroblasts remodel extracellular matrix (ECM) at the pre-metastatic niche, a tumor growth-favoring microenvironment developed in the absence of neoplastic cells^13–15^, secreting inflammatory cytokines and growth factors such as CXCL12, TGF-β, S100A4, and expressing fibronectin and matrix metalloproteinases (MMPs)^16^. Tumor-secreted factors regulate the expression of molecules such as S100A8, S100A9, LOX, fibronectin, MMP-9, MMP-2^17^ that promote the recruitment of specific bone marrow-derived cells (VEGFR1+, CD11b+, CD34+), myeloid cells (CD11b+) as well as differentiated innate and adaptive immune cells^17,18^. In the ECM, CXCL12 interacts with heparin sulfates^19^ and co-localizes with hyaluronic acid (HA)^20–22^; hematopoietic progenitor cells that migrate on HA toward a gradient of CXCL12 acquire spread and polarization expressing CD44 receptor mainly at the leading edge^22,23^. Hyaluronic acid (HA, or hyaluronan) is a glycosaminoglycan which consists of regular repeating non-sulfated disaccharide units of glucuronic acid and N-acetyl glucosamine that exhibits no tissue or species specificity^24^. HA is a principal component of the extracellular matrix, interacts with specific cell surface receptors (CD44, RHAMM and ICAM-1) and contributes to cell–cell adhesion, migration, proliferation and differentiation^25^^,^^26^. Injectable hyaluronic acid (HA) gels such as Intense Belotero® dermal filler are used in aesthetic medicine and thought to be applicable as injectable drug delivery systems^26^. CXCL12, cationic chemokine, binds to glycosaminoglycans (GAGs) through ionic interactions between basic amino acid residues and acidic groups along the disaccharide backbone^27^. Since CXCL12 is highly unstable and rapidly inactivated by CD26, several drug delivery systems have been developed to improve its in vivo residence time^28^. Polymeric hydrogels can hide and protect chemokines from enzymatic cleavage and glycosaminoglycans (GAGs) immobilize and enhance chemokines local concentrations, promoting oligomerization and improving of presentation to the receptors^29,30^. HA, abundant in the bone marrow where CXCL12 is expressed, serves as an anchoring molecule for breast metastatic cells homing through the CD44 receptor^22,31^ Taking advantage of CXCR4 expression on metastatic, aggressive cells we speculate that an artificial tool loaded with CXCL12 might establish a condition similar to pre-metastatic niche that with recruitment of neutrophils and bone marrow derived cells will attract B16-hCXCR4 cells diverting them from secondary metastatic sites. Herein a commercially available dermal filler, HA-based gel, loaded with CXCL12 reproduced a “fake” pre-metastatic niche. The efficacy of this tool was evaluated in (i) recruiting circulating cells to be biologically characterized; (ii) trapping tumor cells and consequently reducing metastases.

## Materials and methods

### Cell culture

B16 cells were transfected with 5 µg of pCMV6-AC-GFP Vector (TrueORF clone; Origene) or with 5 µg of pCMV6-AC-h-CXCR4-GFP Vector (TrueORF clone; Origene). B16-GFP and B16-hCXCR4-GFP murine melanoma cells were grown at 37 °C in 5% CO2 in IMDM with 10% FBS and 2 mM glutamine, 50 µg/mL penicillin, 50 µg/mL streptomycin and 2 mg/mL G418. B16-hCXCR4-GFP cells were stained with Cell Tracker Green CMFDA at concentration of 10 mM (10.8 µl, anhydrous DMSO). Cell Tracker Green CMFDA solution (1 µl) was added to the cells suspended in serum-free medium (4 ml) and incubated for 30 min at 37 °C (Life Technologies). Cell line was morphologically identified monthly.

### Gel preparation

The commercially available HA based gels (Belotero Intense®) were purchased by Merz Pharma. HA fillers were commercially packaged in sterile ready-to-go syringes and appear as a clear, colorless, low-viscosity gels. The recombinant human/ CXCL12/SDF‑1 alpha (R&D System) (300 ng/ml) in PBS/ 0.5% BSA was dropped onto the sterile gel, gently mixed by using a micropipette without introducing bubbles and immediately used. For in vivo experiments, CXCL12 (5 µg/ml) in 100 µl was dropped onto the sterile gel (1 ml) and immediately injected in the subcutaneous flank of C57BL/6 mice.

### Migration

B16-GFP and B16-hCXCR4-GFP cells (2x10^5^/well) were seeded on 24-well transwell inserts (8 µm pore-size) in culture medium containing 0.5% BSA with or without the CXCR4 antagonist AMD3100 (Sigma) (10–100 nM), anti-CXCR7 (11G8, R&D System) (10 µg/ml). Cells were allowed to migrate for 18 h toward BSA 0.5%, CXCL12 (300 ng/ml), CXCL12 (300 ng/ml) loaded in gel (CLG), and empty gel (EG). Some migrations were evaluated at later time points (3, 7, 14, 21, 28 days). The cells were fixed in 4% (w/v) paraformaldehyde in PBS and stained for 15 min with DAPI. Cells that had migrated to the bottom side of the membrane were visualized under the fluorescent microscope (Carlo Zeiss, Axio Scope.A1) and counted (cells in five randomly chosen visual fields). Migration was reported as migration index, the number of cells migrating toward CXCL12 or CLG /number of cells migrating toward BSA 0.5% medium or EG. The cells migrating inside the gels were assessed by fluorescence microscopy (Carlo Zeiss, Axio Scope.A1).

### In vivo metastases assay

CXCL12 (5 µg/ml)-loaded gels (CLGs) and empty gels (EGs) were injected in the subcutaneous flank of 18 (9/group) 6–8-week-old female C57BL/6 mice (Harlan). Five days later 5 x 10^5^ B16hCXCR4-GFP cells were inoculated into the tail vein. The mice were divided into three groups: (I) control mice (no gels) (6/group), (II) mice inoculated with empty gels (EG) (9/group), (III) mice inoculated with CXCL12-loaded gels (CLG) (9/group). Mice were sacrificed 22 days post-cell injection; lungs and gels were analyzed. Gels were digested with hyaluronidase (HAase, Bioindustria) (100 Units/ml) in 4 Units/ml in PBS. Lungs were fixed in 10% buffered formalin, paraffin-embedded and hematoxylin/eosin stained. Microscopic analysis was conducted with a computer-assisted image measurement program by a microscope (BX51 microscope and DP-1 microscope digital camera; Olympus Japan). On days 10 and 22 post cells injection mice were anesthetized with pentobarbital (70 mg/kg) and subjected to retro-orbital blood sampling. Blood was collected using 1 ml syringes with 10 μl fresh EDTA (Spinreact) and processed for detection of the circulating tumor cells (CTCs). The Istituto Nazionale Tumori, IRCCS, Fondazione Giovanni Pascale Independent Ethical Committee approved the study and experiments were performed in accordance with the Institutional Animal Care and Use Committee guidelines.

### Gels (CLG/EG) characterization

CXCL12 (5 µg/ml)-loaded gels (CLGs) and empty gels (EGs) were injected in the subcutaneous flank of 24 (12/group) 6–8-week-old female C57BL/6 mice (Harlan). The mice were sacrificed at: 24 h after gel implantation, before B16-hCXCR4-GFP cells inoculation; at 72 h and 21 days post B16-hCXCR4-GFP cells inoculation. Hydrogels were fixed in 10% buffered formalin, paraffin-embedded as previously described^32,33^, sectioned into 3-µm slices and stained with haematoxylin/eosin. Gels infiltrating cells were quantified as cells/mm^2^ by in 5 consecutive high-power field (HPF) with 400x magnification (0.237 mm^2^/field), using an Olympus BX51 microscope (Olympus, Tokyo, Japan). Slides were incubated with the following primary Abs by overnight incubation at 4 °C: Rabbit polyclonal to anti-mouse CD11b (E-AB-70017, dilution 1:300, Elabscience with heat-induced epitope retrieval (HIER) pH9 pre-treatment); Rabbit polyclonal to anti-mouse CD11c (E-AB-70016, dilution 1:200, Elabscience with heat-induced epitope retrieval (HIER) pH6 pre-treatment); rat monoclonal anti-mouse F4/80 (clone CI:A31, dilution 1:100, Biorad with HIER pH6 pre-treatment); rat monoclonal anti-mouse Ly6G (clone 1A8, dilution 1:1000, BD Becton, Dickinson and Company, with HIER pH6 pre-treatment) polyclonal biotinylated rabbit anti-rat Ig (Dako E0468 1:400 for 60 min at RT) were used as a secondary Ab. UltraVision™ Detection System: HRP Polymer/AEC Chromogen (ThermoScientific) was used for visualization of staining according to the manufacturer’s instructions. The infiltrated cells were scored according to H-score (percentage of cells with staining X intensity value scored from 0 for “no signal” to 3 for “strong signal”)^34^ The evaluation of stained immune cells was performed in duplicate blindly by three independent observers.

### Flow cytometer analyses

Tumoral cells recovered from gels were stained with a PE-hCXCR4 and APC anti-mouse CD45 Ab (BD Biosciences) and analyzed with a FacsDiva software 8.18 (BD Bioscience). The cells were stained with a viability dye 7-AAD to identify living and dead cells. The percentage of CXCR4 positive/ GFP positive cells was evaluated on live cells, 7-AAD negative / CD45 negative cells gate. Single stained samples were performed for compensation controls, and isotype control was used to determine the level of non-specific binding. Red blood cells in 100 μl murine blood cells were lysed and remaining cells were stained with human CXCR4 antibody (clone 11G5), PE-hCXCR4, and APC anti-mouse CD45 Ab (clone 30-F11, BD Biosciences). Also, EG/CLG hydrogels were recovered and digested with hyaluronidase. Cells were stained using 7-AAD (Thermo-Fisher), APC anti-mouse CD45 Ab (BD Biosciences), PE-Cy7 anti-mouse Ly-6G (clone1A8 BD Biosciences) and AlexaFluor 488 anti-mouse CD11b (clone M1/70, Elabscience). Lymphocytes were characterized as CD45 bright/ SSC low on base of cell scattering characteristics and CD45 expression.

### Cell viability analysis

B16-CXCR4-GFP cells were allowed to migrate onto gels at 3, 7, 14, 21 and 28 days. Gels were digested with hyaluronidase as above described, cells recovered and dissociated with 0.05% trypsin/EDTA. Cell suspension was incubated with 10 µL of trypan blue solution and live cells counted on hemocytometer.

### Immunofluorescence

Cells (1 x 10^4^) were seeded on glass coverslips, fixed with 4% paraformaldehyde (15 min, 4 °C) and stained with mouse anti-human CXCR4 primary antibody (clone 12G5 R&D), rabbit anti-cKit primary antibody, mouse anti-αSMA primary antibody, mouse anti-S100 primary antibody and mouse anti-Melan A primary antibody; Alexa Fluor goat anti-mouse 594-conjugated secondary antibody, or Alexa Fluor goat anti-rabbit 488-conjugated secondary antibody, sequentially. DAPI was used to stain the cell nucleus (Carlo Zeiss, Axio Scope.A1).

### Real time PCR

Total RNA from murine blood (100 ul) was extracted using QIAamp RNA Blood Mini Kit (Qiagen), according to the manufacturer’s instructions. DNase-treated RNA (100 ng) was reverse transcribed by Superscript II RNase H-reverse transcriptase according to the manufacturer’s instructions (Invitrogen-Life Technologies, Carlsbad, CA, USA). Real time-PCR was carried out using about 12.5 ng of cDNA in 25 µl final of SYBR Green reaction mixture. An CFX96 Touch™ Real-Time PCR Detection System (BioRad) robocycler was used for the amplification. For turbo-GFP, cycling conditions of the PCR were as follows: initial denaturation (10 min at 95 °C) followed by 40 cycles of denaturation (10 s at 95 °C), annealing (30 s at 58 °C) synthesis (30 s at 72 °C). The gene-specific primers used for the amplification were as follows: mc-kit 5′-TCATCGAGTGTGATGGGAAA-3′ (forward), 5′- GGTGACTTGTTTCAGGCACA-3′ (reverse); mαSMA 5′- TGACCCAGATTATGTTTGA-3′ (forward), 5′- GCTGTTATAGGTGGTTTCG- 3′ (reverse); mβ actin 5′- AGGCTGTGCTGTCCCTGTAT-3′(forward), 5′- AAGGAAGGCTGGAAAAGAGC-3′ (reverse); Turbo GFP 5′-AGGACAGCGTGATCTTCACC-3′ (forward), 5′- CTTGAAGTGCATGTGGCTGT-3′ (reverse); mGAPDH 5′-TCTCCAGGCGGCACGTC-3′ (forward); 5′-TGGCCTTCCGTGTTCCTACCC-3′ (reverse). Subsequently, TurboGFP mRNA was quantified comparing its expression to mGAPDH mRNA levels. Samples were run in triplicate.

### CXCL12/SDF-1α plasma concentration

Blood samples were obtained from retroorbital sinus of anesthetized mice in EDTA and stored at −70 °C until assayed. Mouse SDF-1α was evaluated with CXCL12α Sandwich- ELISA kit (e-Bioscience).

### Statistical analysis

Student’s *t*-tests were used to compare the means of two groups when the assumptions of normality assessed with the Shapiro-Wilks test are met. For independent (unpaired) groups which are non-normally distributed, the non-parametric Mann-Whitney *U* test was used. The non-parametric Kruskal-Wallis test, used in the in vivo experiments, evaluated the significance of the differences of the mean ranks, owing to a lack of compatibility to the normal distribution. Per-comparison two-sided *p* values less than 0.05 were considered statistically significant. The values given are means ± standard deviation (SPSS statistics).

## Results

### CXCL12 loaded gel (CLG) attracted CXCR4 positive cells

**Fig. 1:**
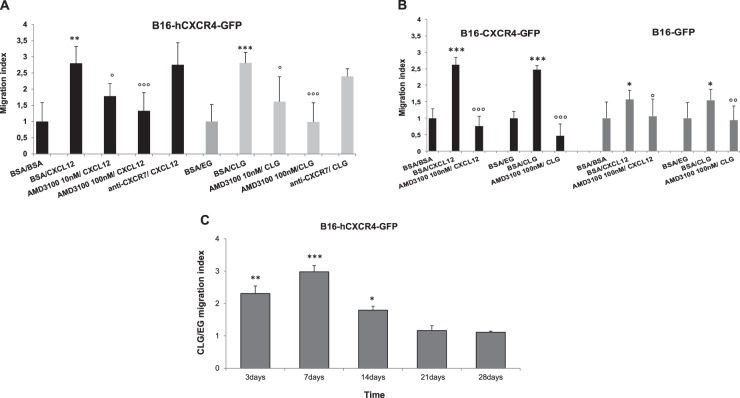
CXCL12 loaded gel (CLG) attracted CXCR4 positive cells. **A** Migration of B16-hCXCR4-GFP cells Cell Tracker Green toward CXCL12 in culture medium or CLG (300 ng/ml) with AMD3100 (10–100 nM) or anti-CXCR7 (10 µg/ml). The results are expressed as index (B16-hCXCR4-GFP cells migrated toward CXCL12 or CLG/ B16-hCXCR4-GFP cells migrated toward BSA (0.5% BSA in culture medium) or EG). Each column represents the mean ± S.D. (*n* = 3). Statistical significances were calculated by Mann-Whitney U test. **p < 0.01 CXCL12 vs BSA or ***p < 0.001 CLG vs EG; °*p* < 0.05; °°°*p* < 0.001 AMD3100 vs CXCL12 or AMD31000 vs CLG. **B** Migration assay of B16-hCXCR4-GFP and B16-GFP cells. B16-hCXCR4-GFP cells and B16-GFP were exposed to the chemokine CXCL12 in culture medium or CLG (300 ng/ml) with AMD3100 (100 nM) in a transwell culture plate. The results are expressed as index. Each column represents the mean ± S.D. (*n* = 3). Statistical significances were calculated by Student’s *t*-test. In B16-hCXCR4-GFP cells ***p < 0.001 CXCL12 vs BSA or CLG vs EG; °°°*p* < 0.001 AMD3100 vs CXCL12 or AMD31000 vs CLG; in B16-GFP *p < 0.05 CXCL12 vs BSA or *p < 0.05 CLG vs EG; °*p* < 0.05 AMD3100 vs CXCL12 or °°*p* < 0.01 AMD31000 vs CLG. **C** Long term migration of B16-hCXCR4-GFP cells Cell Tracker Green toward CLG (300 ng/ml). The results are expressed as B16-hCXCR4-GFP migrated toward CLG/ B16-hCXCR4-GFP migrated toward EG for 3, 7, 14, 21, 28 days. Each column represents the mean ± S.D. (*n* = 3). Statistical analysis of the difference between cells migrated toward CLG and EG at same time point were calculated by Student’s *t*-test. ***p < 0.001; **p < 0.01; *p < 0.05 CLG vs EG

With the intent to attract CXCR4 expressing neoplastic cells a commercially available HA based gel (Belotero Intense®) loaded with CXCL12 was developed (CXCL12-loaded gel; CLG /empty gel; EG). B16-hCXCR4-GFP cells strongly express human CXCR4 (Supplemental Fig. 1A) and, when injected intravenously, develop lung metastases^35^. The B16-hCXCR4-GFP cells were stained with Cell Tracker Green and allowed to migrate toward medium containing CXCL12 (300 ng/ml) or CLG loaded with 300 ng/ml CXCL12. In Fig. 1A, B16-hCXCR4-GFP cells migrated toward CLG with a rate similar to that obtained with soluble CXCL12 (Migration Index respectively, 2.81 ± 0.32 *vs* 2.80 ± 0.52). Migration was specifically inhibited by AMD3100 (Fig. 1A), the unique CXCR4 antagonist clinically approved^36^ but not inhibited by anti-CXCR7 (clone 11G8) suggesting that CXCR7^37^ is not involved in B16-hCXCR4-GFP CXCL12-induced migration. Anti-CXCR7 (clone 11G8), as the small molecule CXCR7 inhibitor CCX771^38^, was highly selective for CXCR7, specifically inhibited CXCL12-dependent migration and competed with CXCL11/CXCL12 binding in CXCR7 expressing MCF-7. (Supplemental Fig. 1B). B16-hCXCR4-GFP migration was also compared to B16-GFP cells (hCXCR4 negative) migration. In Fig. 1B, B16-hCXCR4-GFP cells migrated more efficiently than B16-GFP towards CXCL12 or CLG; B16-GFP cells comparably migrated toward CXCL12 (Migration index, 1.57 ± 0.27) or CLG (Migration index, 1.54 ± 0.32). Same experiments were conducted with CCRF-CEM, T cell leukemia cells (Supplemental Fig. 2, A) and A498, human renal cancer cells^12^ (Supplemental Fig. 3).

### CXCL12 released from CLG induces long term chemotaxis

To investigate the efficacy of CLG–trapped CXCL12 to induce chemotaxis, the B16-hCXCR4-GFP migration toward CLG or EG was evaluated at longer time points. As shown in Fig. 1C B16-hCXCR4-GFP cells specifically migrated toward CLG as compared to EG for 14 days. At 21 and 28 days there were no detectable differences in migration toward CLG or EG suggesting CXCL12 exhaustion. Thus CXCL12 embedded into the gel is able to attract CXCR4 expressing cells. These data were confirmed by the slow in vitro CXCL12 release from CLG [5.43% of the total loaded cytokine released after 7 day incubation (up to 100 h) (data not shown)].

### B16-hCXCR4-GFP cells trapped in EG or CLG gels progressively die

**Fig. 2:**
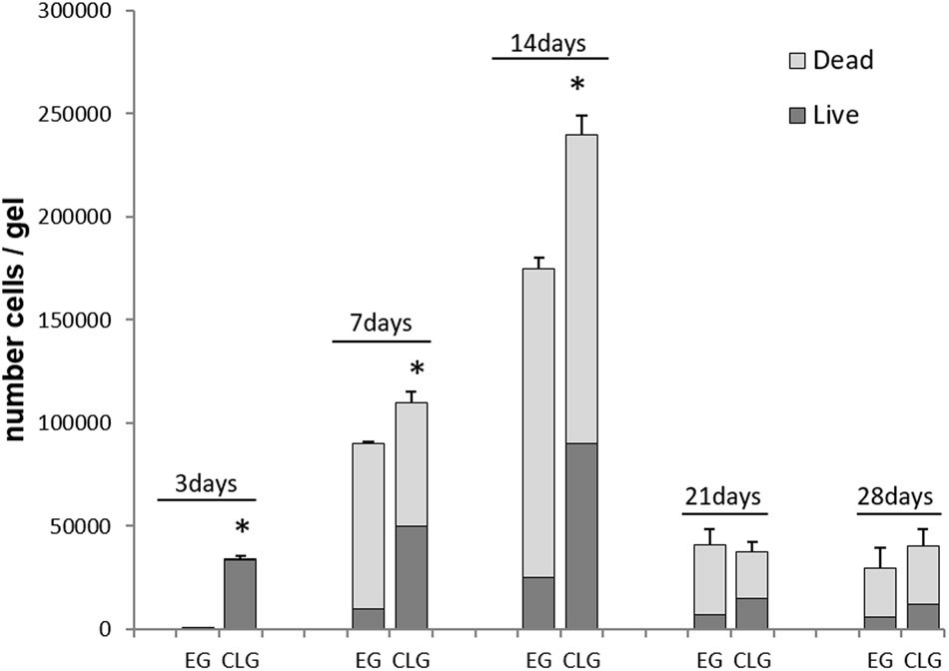
B16-hCXCR4-GFP cells trapped in EG or CLG gels progressively die. In vitro transmigration assay: live /dead B16-hCXCR4-GFP cells migrated into EG or CLG for 3, 7, 14, 21, 28 days. Each column represents the mean ± S.D. (*n* = 3). Statistical significances were calculated by Student’s *t*-test. *p < 0.05 CLG vs EG

To evaluate the viability and the growth capability of B16-hCXCR4-GFP trapped cells, the gels were digested and cells recovered. In Fig. 2, trypan-blue exclusion test demonstrated that recovered cells were viable at three days. In CLG, on day 14 approximately 30% of live cells and 70% of dead cells were detected while in EG the percentage of live cells was 14% and dead cells was 86%. After four weeks the majority of cells were dead in all gels.

### CLG in vivo captured Circulating Tumor Cells (CTCs)

**Fig. 3:**
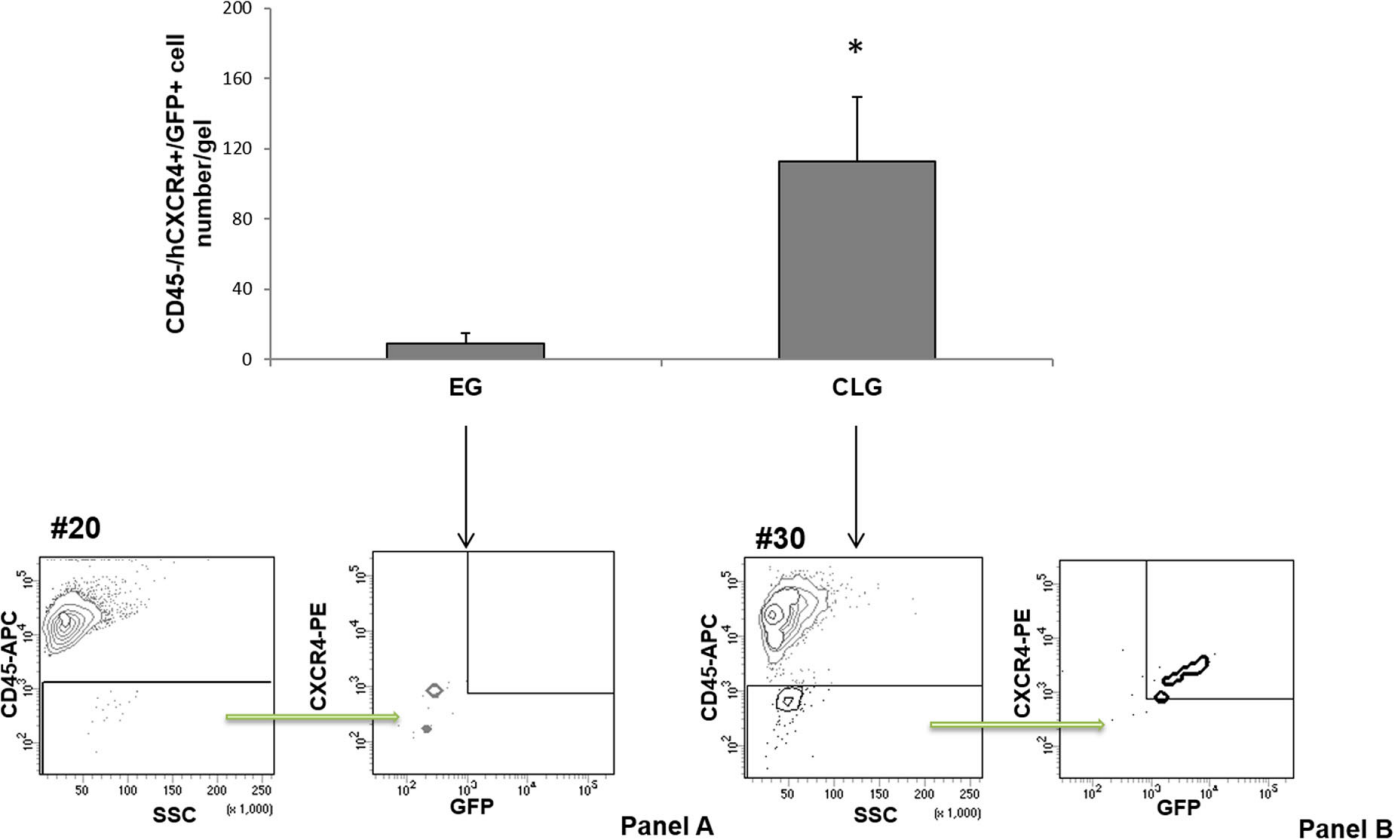
CLG in vivo captured Circulating Tumor Cells (CTCs). Upper panel. B16-hCXCR4-GFP tumor cells (CD45 neg/hCXCR4 plus/GFP plus) isolated from EGs (9 mice) and CLGs (9 mice) at day 22 post-tumor cells inoculation analyzed via flow cytometry. *p < 0.05 as determined by Mann-Whitney U test. Results represent two independent experiments. Lower panel. Representative flow cytometry plots of cells isolated from one EG (**Panel** **A**) and one CLG (**Panel** **B**)

To investigate the efficacy of CLG in attracting CXCR4 positive circulating tumor cells, the syngeneic model of melanoma lung metastasis was employed. CLG [CXCL12 (5 µg/ml) in 100 µl] was subcutaneously injected into the right flank of C57BL/6 female mice. Five days later B16-hCXCR4-GFP cells (5 x 10^5^)^39,40^ were intravenously injected into the mice tail vein. Twenty-two days later mice were euthanized. The gels were recovered, digested and cancer cells characterized as CD45 neg /hCXCR4 plus/ GFP plus cells. The number of CD45neg /hCXCR4plus /GFPplus cells recovered from CLGs was significantly higher than the number of cells isolated from EG (120 ± 36.7 in CLG *vs*16 ± 6.3 in EG) (Fig. 3, upper panel). In Fig. 3 (lower panel) representative dot plots for CD45neg /hCXCR4plus /GFPplus cells isolated from EG (panel A) and CLG (panel B) are shown.

### CLG reduced tumor burden in solid organs

**Fig. 4:**
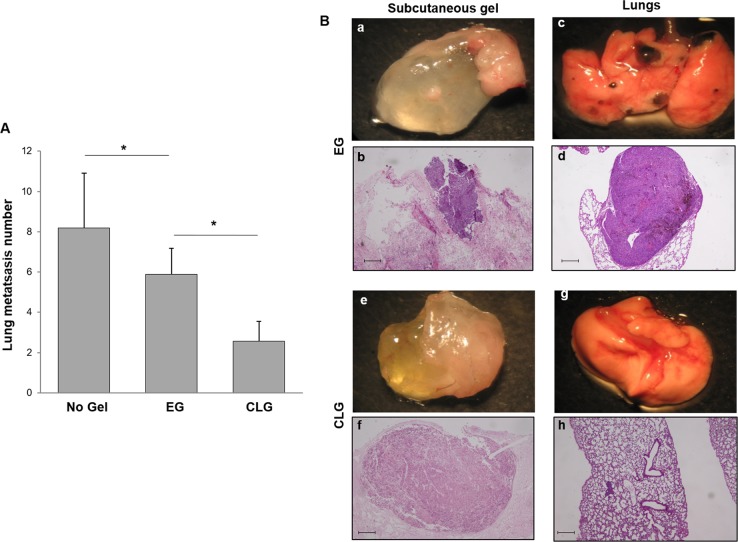
CLG reduced tumor burden in solid organs. **A** Lung metastases number in mice injected with EG (9 mice) versus CLG (9 mice) versus no gel (6 mice). Kruskal-Wallis test were used for statistical analyses. Each column represents the mean ± S.D. *p < 0.05. **B** Representative EG, macroscopic and microscopic evaluation (**a**, **b**), corresponding lungs macroscopic and microscopic evaluation (**c**, **d**). Representative CLG, macroscopic and microscopic evaluation (**e**, **f**), corresponding lungs macroscopic and microscopic evaluation (**g**, **h**). Areas of metastatic lesion are shown at higher magnification (×50). Scale bar is 200 µm. Results represent two independent experiments

To investigate the impact of gel trapped cells in secondary lung colonization, lung metastasis were evaluated. Lung analysis confirmed metastases reduction in mice carrying CLG versus EG (2.5 ± 1.3 versus 5.9 ± 1 metastatic lesions per section) (Fig. 4A). In Fig. 4B representative recovered lungs and corresponding subcutaneous gels were shown (EG; a-d, and CLG; e-h). A higher number and larger size of lung metastasis was detected in mice carrying EG (Fig. 4B, panel c, d), as compared to mouse carrying CLG (Fig. 4B, panel g, h). Conversely, although EG and CLG trapped cells developed metastasis-like lesions (Fig. 4B, panel a, b, e, f), the nodules developed within the EG (Fig. 4B, panel b) were smaller (Fig. 4B, panel f).

### CLG is infiltrated by inflammatory cells

**Fig. 5:**
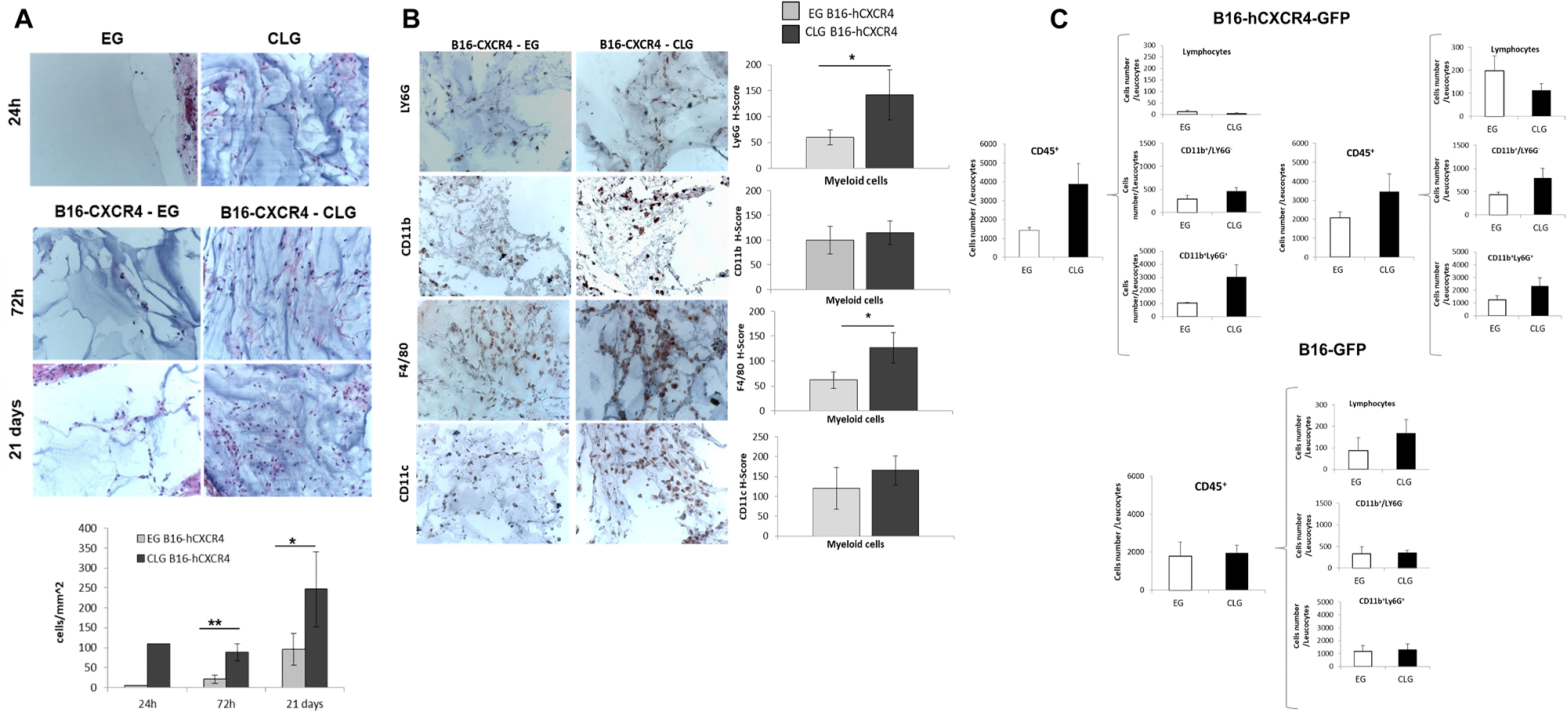
CLG is infiltrated by inflammatory cells. The EG/CLG collected from mice necropsy at 24 h before-B16-hCXCR4-GFP cells inoculation and 72 h and 21 days post-B16-hCXCR4-GFP cells inoculation. **A** Representative micrograph of surgical derived EG/CLG stained with H&E at each time point (50x magnification). The sections were stained with H&E and hydrogels-infiltrating cells content were quantified as mean cells/mm^2^ ± SD. The bar graphs represent mean values ± SD. The value of *p* < 0.05 was considered significant; Mann-Whitney test *p < 0.05; **p < 0.01. **B** Representative micrographs of surgical derived EG/CLG stained for Ly6G (neutrophils specific marker), F4/80 (macrophages specific marker), CD11b (monocytes/macrophages specific marker) and CD11c (dendritic cells specific marker) antigen (200x magnification). The bar graphs represent mean values ± SD. The value of p < 0.05 was considered significant; Mann-Whitney test *p < 0.05. **C** (Upper panel) Flow cytometric analysis of cells removed from EG and CLG at 72 h (left) and day 21 (right) post B16-hCXCR4-GFP cells injection; Cell populations are reported as cell number of leucocytes: CD45 positive cells; Lymphocytes population; Monocytes CD11b+/Ly6G- cells; Neutrophil CD11b+Ly6G+cells. (Lower panel) Flow cytometric analysis at day 21 post B16-GFP cell line injection of cells removed from gel with-out CXCL12 (EG) and with CXCL12 (CLG). Cell populations are reported as cell number of leucocytes: CD45 positive cells; Lymphocytes population; Monocytes CD11b+/Ly6G- cells; Neutrophil CD11b+Ly6G+cells. The bar graphs represent mean values ± SEM

To analyze the milieu within the gels, infiltrating cells were characterized. EG and CLG were analyzed “in vivo” 24 h post gels implantation, before of tumor cell inoculation, −72 h post cell inoculation and −21 days post cell inoculation. In Fig. 5A higher cell infiltration was detected in CLG as compared to EG already at 24 h post implantation. Higher infiltrate was detected at 72 h and 21 days post B16-hCXCR4-GFP inoculation. As shown in Fig. 5A infiltrating cells efficiently migrated toward CLG at 24, 72 h and 21 days (EG: 0.0 vs CLG: 110 at 24 h, *n* = 2; EG: 21 ± 9.71 vs CLG: 89 ± 21 at 72 h, *n* = 3; EG: 96 ± 39.9 vs CLG: 247 ± 94.9 at 21 days, *n* = 5). Immunohistochemical evaluation of EG/CLGs at day 21 post-B16-hCXCR4-GFP inoculation demonstrated that neutrophils (identified as Ly6G+) and monocytes/macrophages (identified as F4/80+) efficiently infiltrated CLG (Fig. 5B). In Fig. 5C- Upper left panel, at day 3 and Upper right panel, day 21 post-B16-hCXCR4-GFP inoculation flow cytometric analysis demonstrated higher density of CD45-positive leukocytes in CLG compared to EG; in particular, CLG displayed higher number of neutrophils (CD11b+Ly6G+) and myeloid (CD11b+/Ly6G-) cells as compared to EG. CD11b+Ly6G+ and CD11b+/Ly6G- cells have been implicated in pre-metastatic niche^41,42^. Conversely, in the Fig. 5C- Lower panel, CD45-positive leukocytes were comparable in EG or CLG 21 days post-B16-GFP inoculation suggesting that CLG recruited CXCR4 + tumor cells may affect the recruitment of immune cells^41^.

### In vitro characterization of CLG/EG recovered cells

**Fig. 6:**
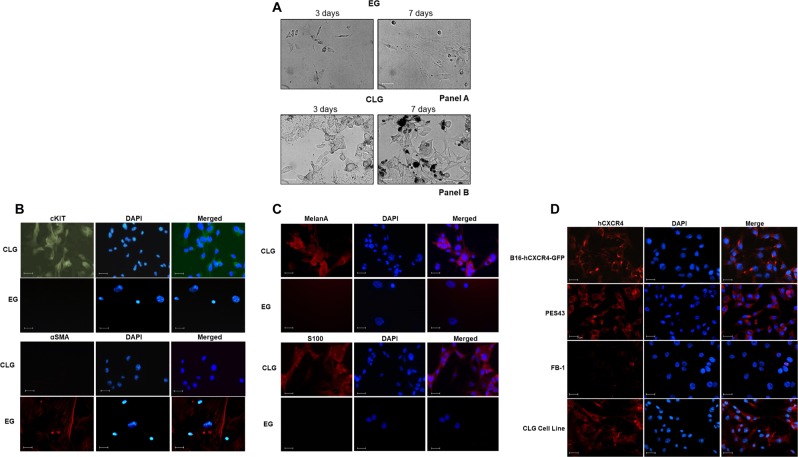
In vitro characterization of CLG/EG recovered cells. **A** Cells recovered from EG and CLG gel were seeded on plates immediately after collection. Microphotographs from EG (panel A) and CLG (panel B) at 3 and 7days (Axiovert10 Microscopy-Zeiss). Scale bar is 20 µm. CLG cells express **B** c-Kit, **C** Melan A, S100 and **D** CXCR4 while EG cells express **B** α-SMA but do not express c-Kit, Melan A and S100. Cells (1 x 10^4^) were seeded on glass coverslips, fixed with 4% paraformaldehyde (15 min, 4 °C) and stained with rabbit anti-c-Kit primary antibody, mouse anti-αSMA primary antibody, mouse anti-S100 primary antibody mouse, mouse anti-Melan A primary antibody and mouse anti-human CXCR4 primary antibody (clone 12G5 R&D). DAPI was used to stain the cell nucleus (Carlo Zeiss, Axio Scope.A1). CXCR4 expression was compared to PES43, human melanoma cell line-CXCR4 expressing, FB-1, human anaplastic thyroid cancer cell line- low CXCR4 expressing and murine B16-hCXCR4-GFP cells evaluated in the in vitro/in vivo assay. DAPI was used to stain the cell nucleae. Magnification 400X. Scale bar is 25 µm

Cells recovered from EG and CLG are shown in Fig. 6A at 3–7 days of cell culture. Cells isolated from CLG displayed spherical shape (Fig. 6A, Panel B), stained positive for c-Kit^43^, negative for Alpha-Smooth Muscle Actin (α-SMA) (Fig. 6B), conversely EG recovered cells shaped as fibroblasts (Fig. 6A, Panel A) and stained negative for c-Kit, positive for α-SMA (Fig. 6B). In addition, c-kit and α-SMA expression was evaluated on CLG- and EG- isolated cells through qRT-PCR confirming high expression of α-SMA and low c-kit in EG, and high expression of c-kit and low α-SMA in CLG (Supplemental Fig. 4A). CLG isolated cells appeared epithelial, melanin rich (Fig. 6A, Panel B), express the Melan A^44^ and S100 ^45^ suggesting a melanoma cell line (Fig. 6C) while EG isolated cells appeared negative for Melan A and S100 (Fig. 6C). In Fig. 5D the CLG recovered cells expressed human CXCR4 as compared to the injected cells B16-hCXCR4 cells and to PES43, human melanoma cells. As shown in Supplemental Fig. 4B, CLG recovered cells expressed GFP RNA level as compared with B16-hCXCR4-GFP cells. The CLG isolated cells actively migrated toward CXCL12 and migration is inhibited by AMD3100 (Supplemental Fig. 4C). The ability to isolate tumor cells while subtracting them to conventional sites of metastasis allows isolation and characterization of circulating tumoral cells.

### CTCs in mice carrying CLG/EG

**Fig. 7:**
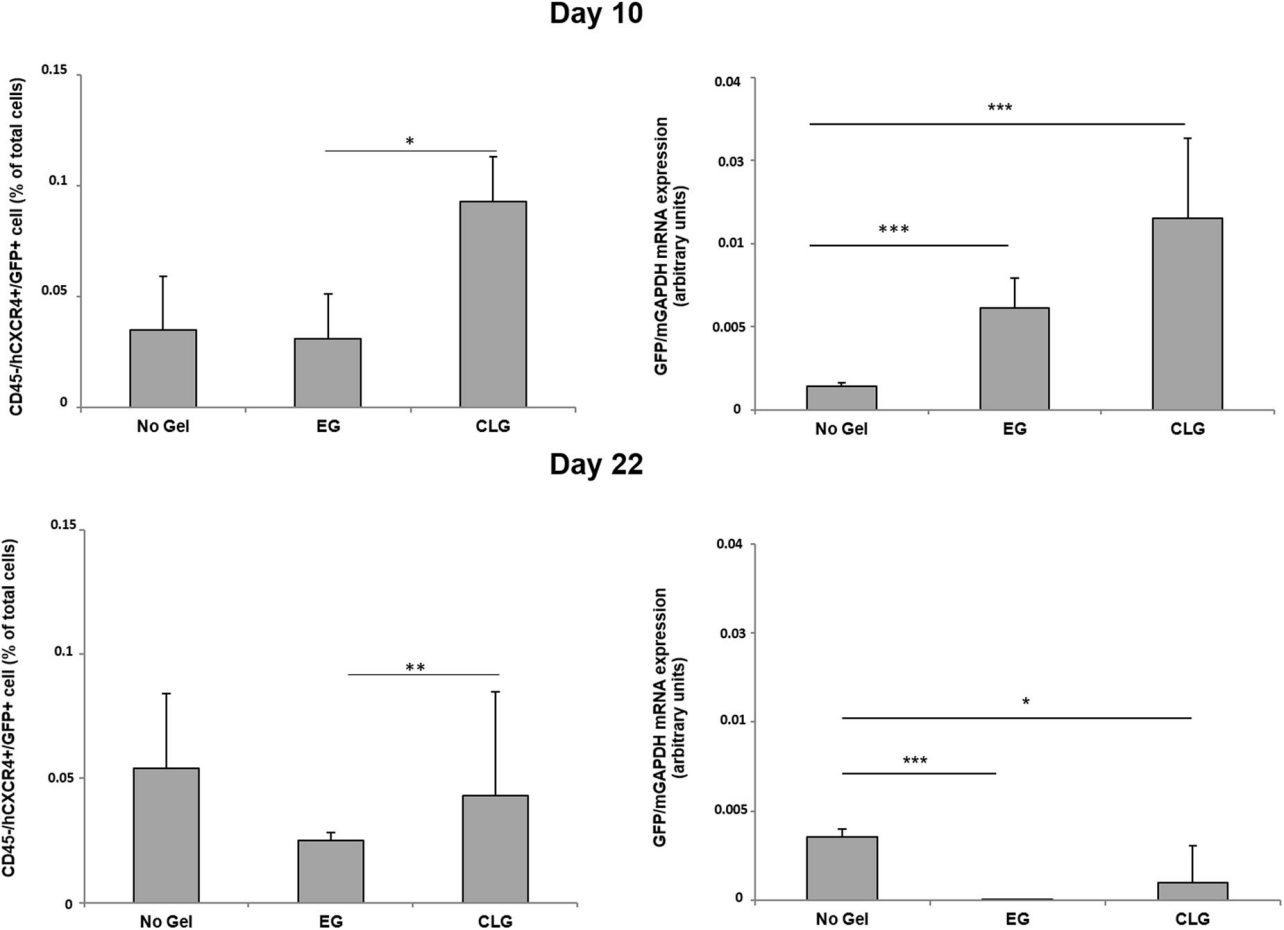
CTCs in mice carrying CLG/EG. Blood CTCs (CD45 neg/hCXCR4 pos/GFP pos) in mice carrying- EG (9 mice) and -CLG (9 mice) versus no gel (6 mice) at day 10 (upper panel) and day 22 (lower panel) post iv B16-hCXCR4-GFP cells injection. CTCs were identified as CD45neg hCXCR4pos GFPpos cells (left) and GFP gene expression (right). *p < 0.05; **p < 0.01; ***p < 0.001as determined by Mann-Whitney *U* test. Results represent two independent experiments

The possible impact of CLG and EG on the number of circulating tumor cells (CTCs) was evaluated at 10 and at 22 days post cell injection. Briefly 100 µl of retrorbital blood was obtained and CTCs identified as CD45 neg/hCXCR4 pos/GFP pos. The number of CTCs isolated in mice CLG carrying was significantly higher compared to CTCs retrieved in mice EG carrying. As shown in Fig. 7 (upper-left panel) higher number of CTCs was detected in the blood at 10 days from mice CLG (0.09 ± 0.02%) versus EG (0.03 ± 0.02%). This difference, although lower, is still significant at 22 days (CLG 0.04 ± 0.01% versus EG 0.02 ± 0.01%) (Fig. 7, lower-left panel). In concordance, GFP mRNA was higher in CTCs isolated from CLG compared to EG carrying mice at 10 and 22 days (respectively, CLG 0.02 ± 0.009% versus EG 0.01 ± 0.003% and CLG 9.7E-04 ± 0.002% versus EG 2.7E-05 ± 2.3E-05%) (Fig. 7, right panel).

### CXCL12 increased in mice carrying CLG/EG

**Fig. 8:**
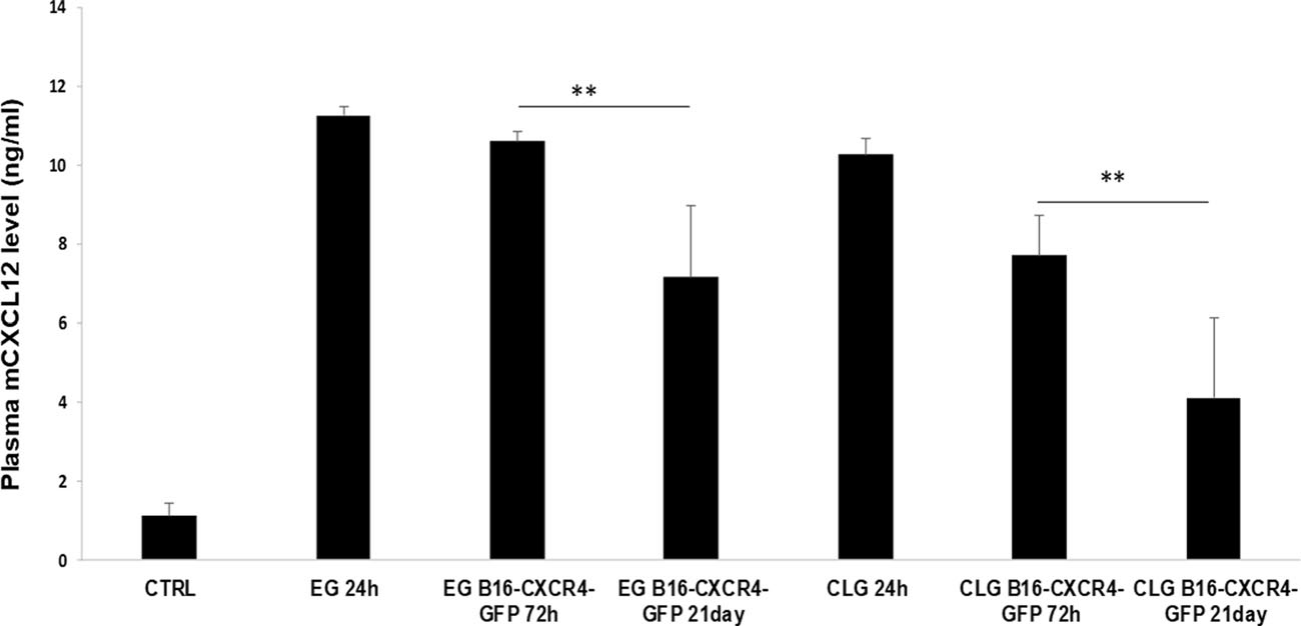
CXCL12 increased in mice carrying CLG/EG. Plasma of mice carrying- EG and -CLG was collected at day 0 from gel-not injected mice, at 24 h after gel inoculation before B16-hCXCR4-GFP cells inoculation; at 72 h or day 21 post cells inoculation and levels of murine CXCL12 were analyzed by ELISA. **p < 0.01, as determined by Student’s *t* test. Results represent two independent experiments

To investigate on possible mechanisms responsible for increased numbers of CTCs in CLG, circulating CXCL12 was evaluated in peripheral blood collected from CLG/EG bearing C57Bl/6 mice. As shown in Fig. 8, both CLG and EG carrying mice displayed higher murine CXCL12 plasma level at 24 h after gel inoculation consistent with inflammatory reaction induced by gel implantation. Circulating CXCL12 at 24 h was 11.3 ± 0.2 ng/ml in mice carryng EG and 10.2 ± 0.4 ng/ml in mice carryng CLG compared to 1.1 ± 0.3 in gel-not injected mice (*p* < 0.01). CXCL12 blood concentration was not statistically different between mice EG or CLG-inoculated at 24 h post gel implantation. In both groups, reduction of blood CXCL12 was observed at 72 h and 21 days post B16-hCXCR4-GFP cells inoculation (at 72 h CXCL12 was 10.6 ± 0.3 ng/ml in EG -mice and 7.7 ± 1.0 ng/ml in CLG- mice while at 21days CXCL12 was 7.1 ± 1.8 ng/ml and 4.1 ± 2.0 ng/ml respectively, in EG and CLG-mice) suggesting that plasma CXCL12 was not influencing the propensity of tumor cell homing to the lungs/CLG.

## Discussion

With the primary intent to collect circulating tumor cells expressing CXCR4, a new device composed of a commercially available dermal filler, hyaluronic acid based gel (Belotero Intense®), loaded with CXCL12 was realized. The CXCL12-loaded gel (CLG) attracted circulating CXCR4 positive melanoma cells diverting them from secondary sites. The cells isolated from CLG mimicked the original injected cells and could be expanded in vitro providing a valid source of tumor cells for further characterization. Moreover, the CLG, capturing CTCs and diverting them from conventional metastatic sites, induced a net decrease in lung metastases. Interestingly, an increased number of circulating tumor cells (CTCs) were observed in CLG bearing mice. Mechanical devices impairing metastases were previously described^39,42,46–48^. To mimic a functional and reproducible bone, silk scaffolds coupled with bone morphogenetic protein-2 (BMP-2) seeded with bone marrow stromal cells (BMSC) were developed^46^. Similarly, a chimeric bone construct was realized with biodegradable tubular composite scaffolds seeded with human mesenchymal progenitor cells and loaded with osteogenic protein-1^47^. Although these examples provided “proof of principle” for a bioengineered humanized model of bone metastasis, the clinical translation is complex. A sort of pre-metastatic niche was developed with a 3D-scaffold embedded with exosomes (M-Trap) capturing human ovarian cancer cells disseminated in the peritoneal cavity^39^. This model demonstrated that an added matrix could segregate metastatic tumor cells in a defined environment such as peritoneal cavity. Micro particles (MPs) containing carcinoma-associated fibroblasts (CAFs), which continuously deposit a pro-adhesive matrix at the surface, selectively bound ovarian neoplastic cells within the peritoneal compartment of the MPs^48^. In a xenograft model of breast cancer an implanted biomaterial (PLG) scaffold recruited circulating metastatic breast cancer cells (MDA-MB-231BR)^42,49^ and human breast cancer brain metastasis derived three-dimensional CAFs aggregates expressed significantly higher levels of CXCL12 and CXCL16 than CAFs aggregates generated from primary breast tumors or normal breast, suggesting that chemokine modulation is crucial in regulating metastasis^1^.

The herein described tool CLG represents an innovative and simple solution to trap circulating tumor cells and, secondarily, to reduce lung metastasis. The main hypothesis herein was that interfering in the process that drives tumor cells from tumor to secondary organs, we may reduce the number of effective cells able to develop metastasis. Metastasis development is a very inefficient process with 0.01% or less of circulating tumor cells able to develop secondary tumors^50^. The CXCL12 gel will subtract CXCR4 overexpressing cells and trap them in a pseudo-niche, where the cells will eventually die within 2 weeks. In doing so it might reduce the critical number of metastasis initiating cells. We may also hypothesize that ‘sensing’ of CXCL12 inside the gel increases the “signal noise” attracting CXCR4 positive cells and affecting seeding in secondary organs as demonstrated by the largest number of circulating cells detected in mice carrying CLG. Since the hyaluronic acid of the hydrogel exerts itself an effect of pseudo-niche, we expect that the cells will be trapped also there. This is consistent with the reduction of metastases in EG compared to the number of metastasis developed in mice in absence of gel (5.9 versus 8.2 metastatic lesions per section). Interestingly, recent evidence demonstrated that local delivery of lauroyl-gemcitabine lipid nanocapsule based hydrogel (GemC12-LNC) in the tumor resection cavity of glioblastoma could prevent local recurrence^51^. The CXCL12 loaded hydrogels in adjuvant setting may trap the undetectable, occult and harboring micrometastases cells reducing future recurrences. This is based on the capability of the matrix, dermal filler, plus the properties of CXCL12 to develop a suitable pre-metastatic niche with attractive capability for CXCR4 expressing circulating tumor cells. VEGFR1 + hematopoietic precursor cells, along with fibronectin and associated stromal cells modify the local microenvironment and regulate the homing and retention of hematopoietic precursor cells as well as tumor cells^17,52^. Liu et al. developed a CXCL12 biomimetic tumoral niche with a thin and soft polyelectrolyte film. CXCL12 presentation was spatially controlled at the ventral side of breast cancer cells inducing lamellipodia and filopodia mediated by CXCR4. CD44 acted in concert to drive a specific matrix-bound CXCL12-induced cell response associated with ERK signaling^53^. Herein CLG recruited higher number of cells compared to EG; although at lesser extent, EG was also able to recruit circulating cancer and mesenchymal cells suggesting that the hyaluronate matrix exerts attraction^21,22,54,55^. Ko Cy et al. developed in vivo model to investigate inflammation-mediated cancer metastasis through biomaterial microspheres. Interestingly, metastatic cancer cells B16F10 injected into the peritoneal cavity migrated into subcutaneous microsphere^56^. The immune cells recruitment to implanted biomaterials may be different in tumor bearing animals versus healthy animals^42^. In our manuscript the CXCL12 loaded gel was able to recruit and trap B16-hCXCR4-GFP cells as previously shown^57^. Quantitative and qualitative differences were detected within the CLGs and EGs inflammatory infiltrate. CLG showed higher number of CD45-positive leukocytes mainly neutrophils CD11b+Ly6G+cells, myeloid CD11b+Ly6G- cells and macrophages F4/80 as compared to EG^58^. CXCL12 insists also on CXCR7, which is phylogenetically closely related to chemokine receptors, binds CXCL12 with a higher affinity than CXCR4, but fails to couple with G proteins to induce typical chemokine receptor–mediated cellular responses^59^. Although in our gels CXCR7 antagonism is not able to interfere with migration of CXCR4 expressing cells toward CLG/EGs, it was recently demonstrated that CXCR7 is expressed on CD14^+^CD16^+^ mature monocytes and a small molecule CXCR7 antagonist (CCX771) can prevent CD14^+^CD16^+^ monocyte transmigration into the central nervous system from uninfected and HIV infected individuals^60^. Intriguingly, the number of CTCs isolated in mice carrying CLG was significantly higher compared to CTCs retrieved in mice carrying EG. To better define this phenomenon blood CXCL12 was evaluated but CXCL12 clearly increased in mice gels-inoculated, either empty or CXCL12-loaded consistent with inflammation gels induced. In both groups, EG/CLG-inoculated, reduction of blood CXCL12 was observed at 72 h and 21 days post B16-hCXCR4-GFP cells inoculation suggesting that plasma CXCL12 was not influencing the propensity of tumor cell homing to the lungs/CLG. Bertolini et al. defined lung cancer initiating cells (CICs) as CD133+CXCR4+cells with metastatic potential inhibited by CXCR4 antagonism^61^. In fact CTC enumeration and CXCR4 expression are promising prognostic biomarkers for CTCs in Extensive-Disease Small Cell Lung Cancer (ED-SCLC) at baseline and post-treatment^62^. For patients at risk of recurrence, CLGs would allow characterization of CTCs and praecox recognition of metastasis disease. CTCs isolation is still a high unmet need due to the limited number of cells generally retrieved (1 in 10^9^ red blood cells)^63^ . Injecting 1x10^6^ of mouse breast cancer GFP-Luc-4T1 in tail vein of BALB/c syngeneic mice resulted in detection of 16 Luc-CTCs comparing three CTC detection methods^64^. The patient subcutaneous devices implantation is currently evaluated in clinical trials. Agarose devices containing murine renal cancer cells (RENCA cells) were safely inoculated in patients abdominal cavity. RENCA cells, encapsulated in the agarose gel, selected a stem cell–like subpopulation which drove colony formation in the macrobeads and produced diffusible substances that markedly inhibited in vitro and in vivo proliferation of epithelial-derived tumor cells outside the macrobeads^65^ (NCT02046174). Phase II studies in patients with colorectal, pancreatic or prostate cancers are in progress (NCT01174368, NCT01053013). In conclusion, our study shows that a commercially available dermal filler loaded with CXCL12 is able to capture and divert neoplastic CXCR4 expressing cells. Behind the suggestion of a diminution of the metastatic efficiency, it is possible to speculate that the device will allow identification and characterization of potential metastatic cells. The praecox identification and definition of metastatic cells may enable efficient interventional strategies.

## Supplementary information

Supplemental Data
