# Supplementary material for: CXCL12 loaded-dermal filler captures CXCR4 expressing melanoma circulating tumor cells
Source: Cell Death Dis. 2019 Jul 22;10(8):562. doi: 10.1038/s41419-019-1796-6 (PMC6646345; doi:10.1038/s41419-019-1796-6)
Supplement: Supplementary file 1 — Supplemental Data [file 41419_2019_1796_MOESM1_ESM.docx]

**SUPPLEMENTAL FIGURES**

**
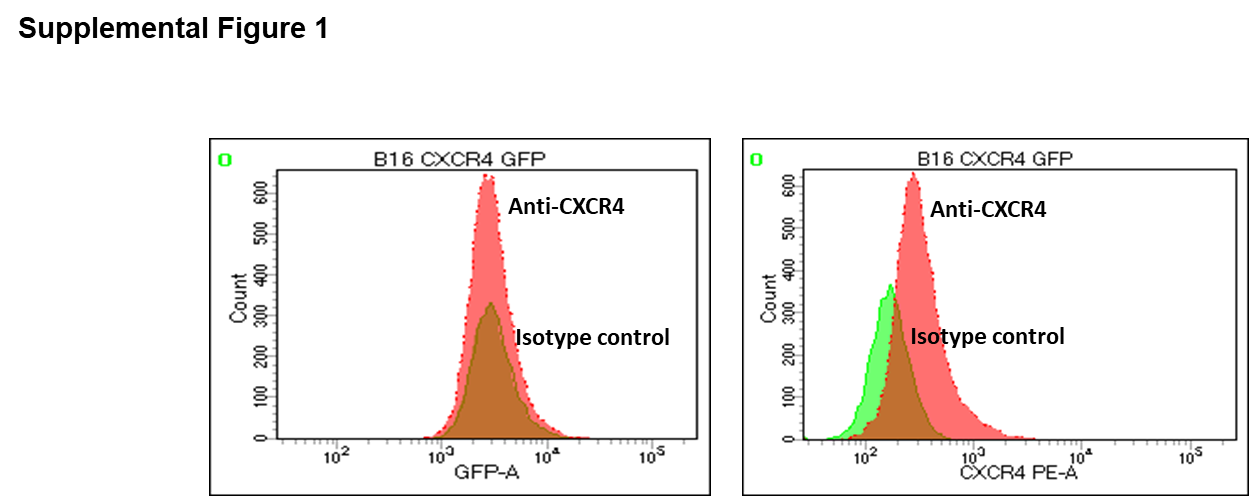
**

**A**

**B**


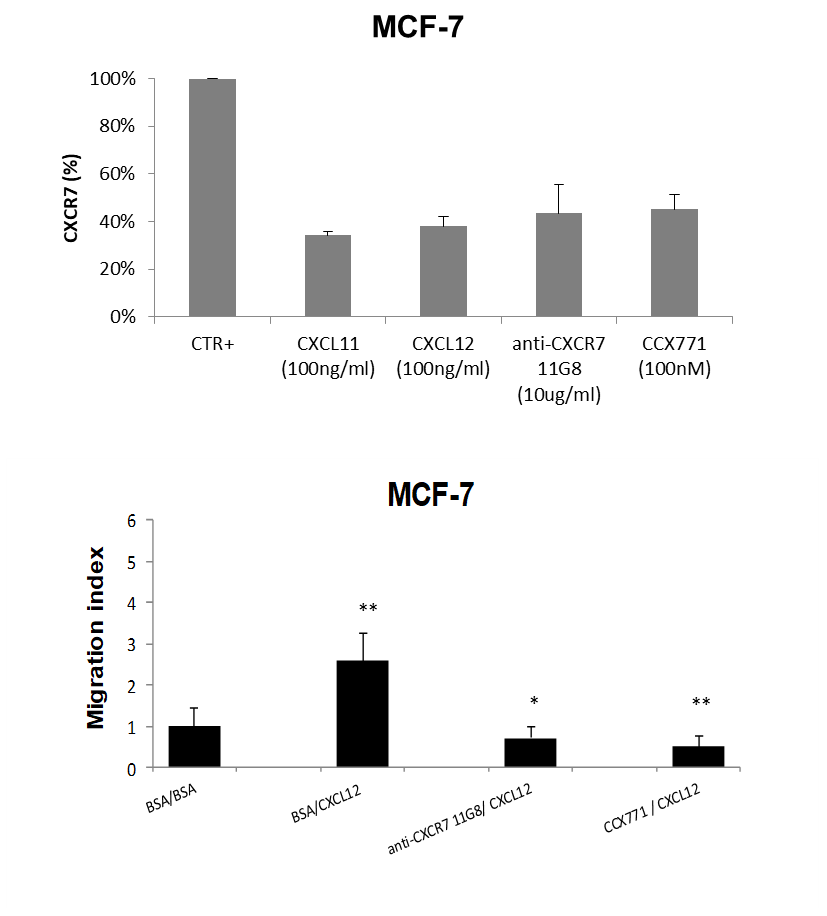


**Supplemental Figure 1.** **CXCL12 loaded gel (CLG) attracted CXCR4 positive cells.** (A) Cell surface expression of hCXCR4 and GFP expression were determined by flow cytometry on transfected B16 murine melanoma cells (B16-hCXCR4-GFP). (B) (Upper). MCF-7 cells were preincubated for 45 minutes with CXCL11 (100ng/ml), CXCL12 (100ng/ml), anti-CXCR7 11G8 (10ug/ml) and CCX771 (100nM). The binding was evaluated by flow cytometry using anti- clone 11G8 anti- CXCR7 antibody (FAB4227A R&D). Data are presented as bar graph showing mean ± SD. (Lower) CXCL12-dependent cell migration in MCF-7 toward CXCL12 (100ng/ml) in the presence of anti-CXCR7 11G8 (10ug/ml) and CCX771 (100nM). The results are expressed as MCF-7 migrated toward CXCL12/ MCF-7 migrated toward BSA. Each column represents the mean ± S.D. (n=3). Statistical significances were calculated by Student’s t-test.


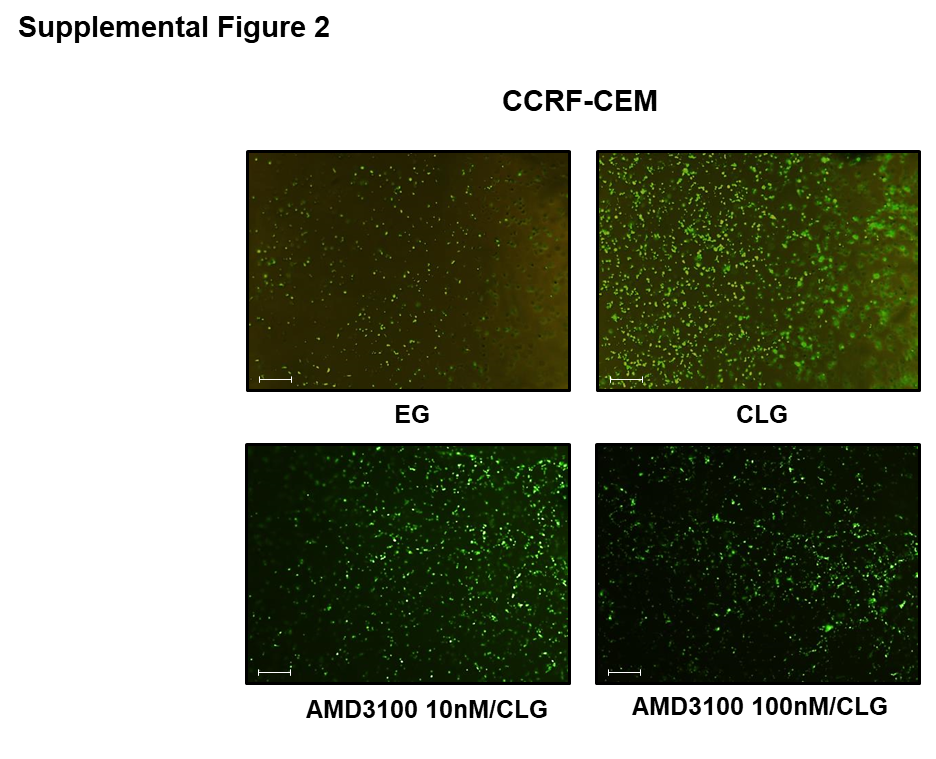


**A**


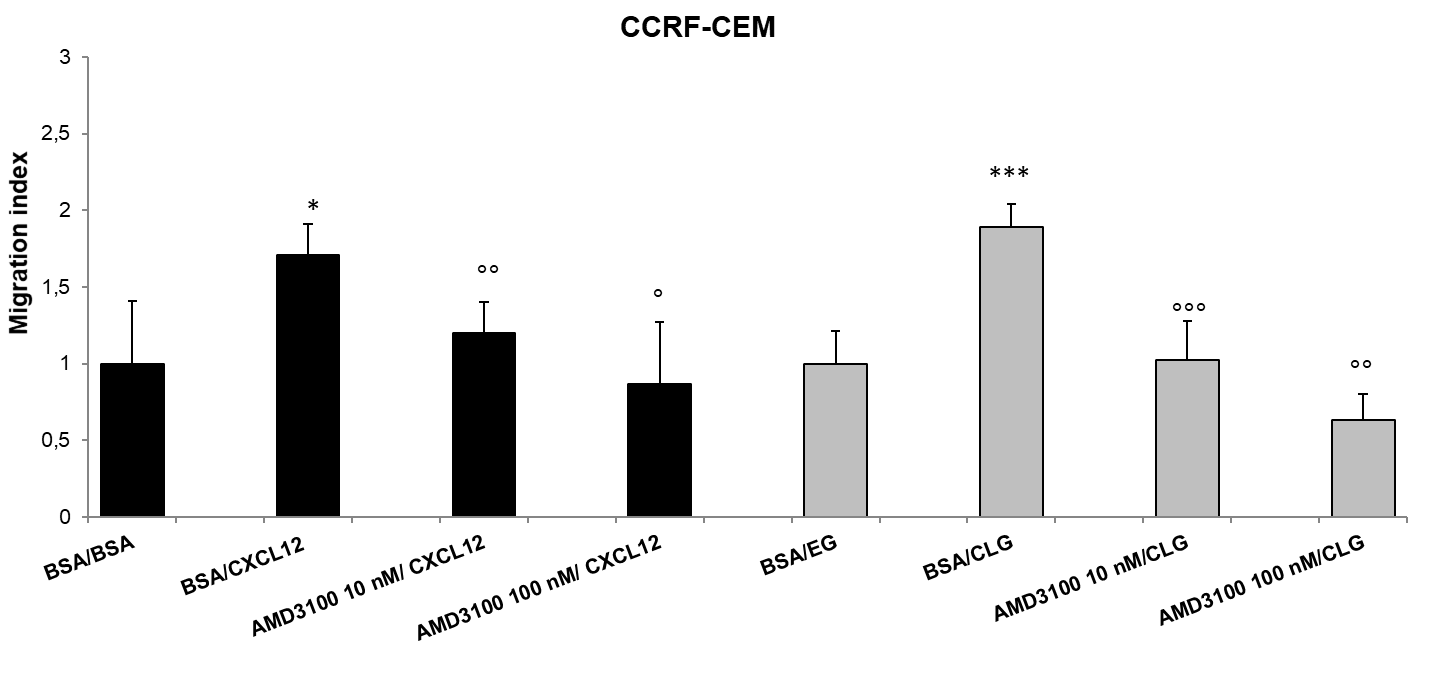


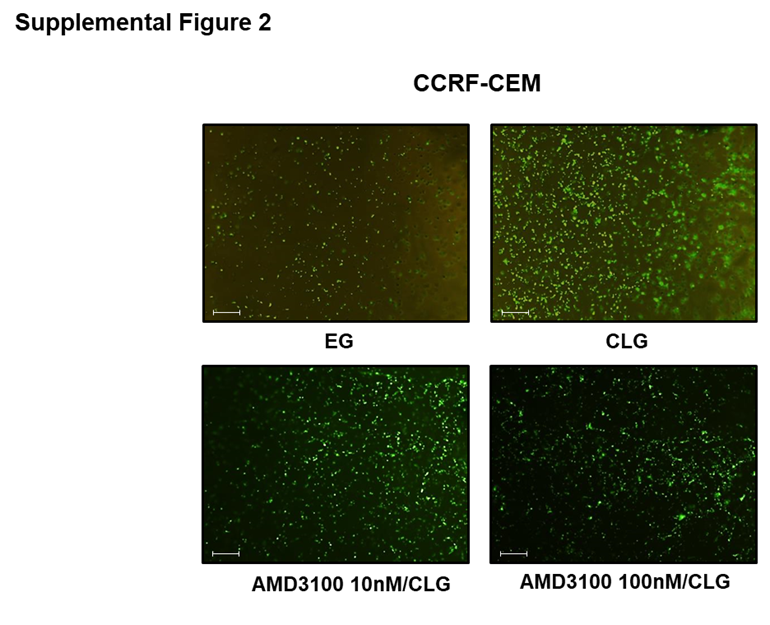


**B**

**Supplemental Figure 2.** **CXCL12 loaded gel (CLG) attracted CXCR4 positive cells.** (A) CXCL12-dependent cell migration in CCRF-CEM toward CXCL12 in culture medium or CLG (300 ng/ml) in the presence of AMD3100 (10-100 nM). The results are expressed as CCRF-CEM migrated toward CXCL12 or CLG/ CCRF-CEM migrated toward BSA or EG. Each column represents the mean ± S.D. (n=3). Statistical significances were calculated by Mann-Whitney U test. * p<0.05 CXCL12 vs BSA or *** p<0.001 CLG vs BSA; °° p<0.01, ° p<0.05, AMD3100 vs CXCL12 or °°° p<0.001, °° p<0.01, AMD31000 vs CLG. (B) Representative images of CCRF-CEM cells Cell Tracker Green migrated inside the EG as compared to CLG and CLG plus AMD3100 at indicated doses. Scale bar is 200 µm.


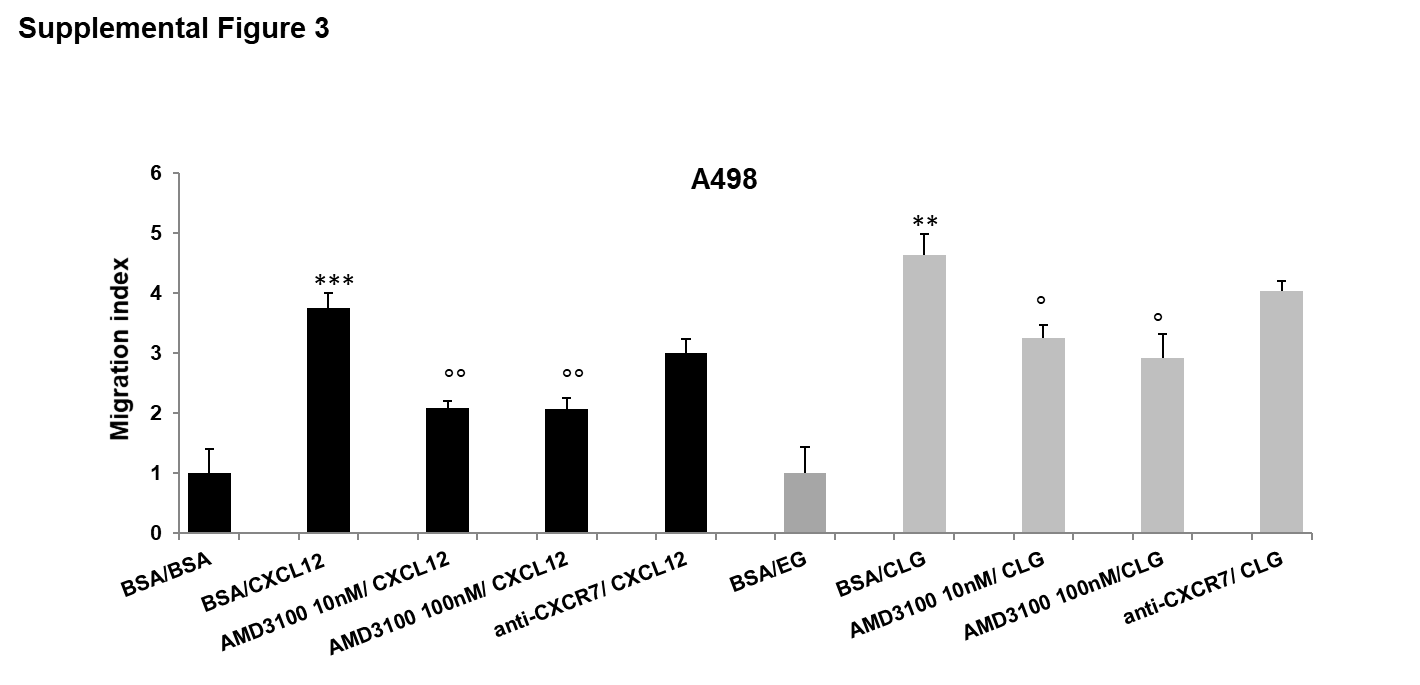


**Supplemental Figure 3. CXCL12 loaded gel (CLG) attracted CXCR4 positive cells.** CXCL12-dipendent cell migration in A498 toward CXCL12 in culture medium or CLG (300 ng/ml) in the presence of AMD3100 (10-100 nM) and anti-CXCR7 (10µg/ml). The results are expressed as A498 migrated toward CXCL12 or CLG/ A498 migrated toward BSA (0.5% BSA in culture medium) or EG. Each column represents the mean ± S.D. (n=3). Statistical significances were calculated by Student’s t-test. *** p<0.001 CXCL12 vs BSA or ** p<0.01 CLG vs EG; °° p<0.01, AMD3100 vs CXCL12 or ° p<0.05 AMD31000 vs CLG.


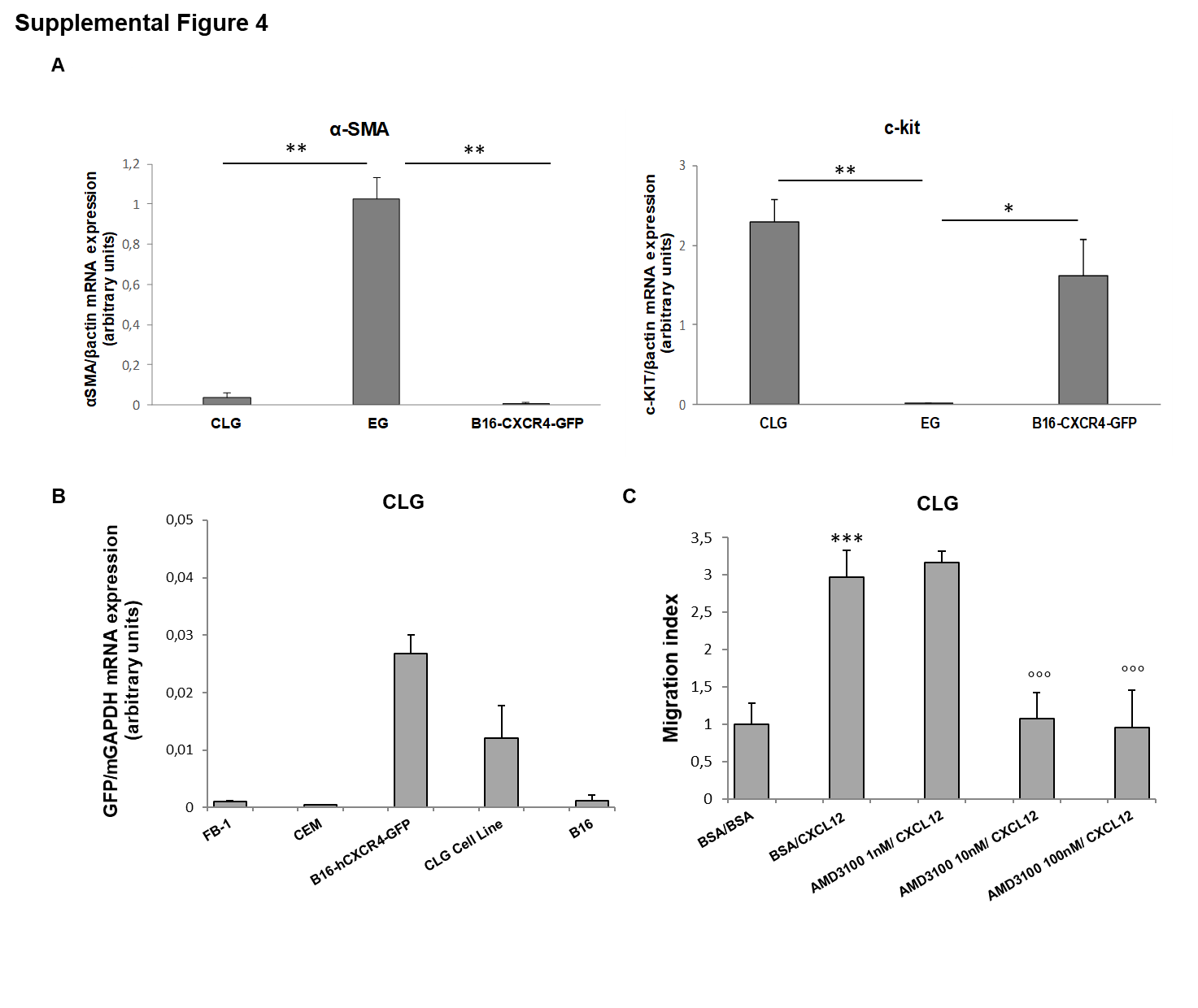


**Supplemental Figure 4. Ex vivo culture of gel trapped cells from CLG**. (A) The mRNA expression levels of α-SMA and c-kit in CLG versus EG cells line by qRT-PCR using β-actin gene as the normalization control. Alpha SMA and c-kit gene expression in CLG and EG cells was compared to alpha SMA and c-kit gene expression in murine B16-hCXCR4-GFP. (B) GFP gene expression in the in CLG cell line was compared to GFP gene expression in murine B16-hCXCR4-GFP, in vivo inoculated cells. FB-1, human anaplastic thyroid cancer cell line, B16, murine melanoma cell line and CEM are used as GFP negative control. (C) CXCL12-dipendent cell migration in CLG cell line in the presence of AMD3100 (1-10-100 nM). Cells migrated toward CXCL12 (300ng/ml) for 18 hours. The results are expressed as CLG cells migrated toward CXCL12/ CLG cells migrated toward BSA (0.5% BSA in culture medium). Each column represents the mean ± S.D. (n=3). Statistical significances were calculated by Student’s t-test. *** p<0.01 CXCL12 vs BSA; °°°p<0.01 AMD3100 vs CXCL12.

**SUPPLEMENTAL METHODS**

*Binding Assay.* To evaluate the specific anti-CXCR7 (clone 11G8) binding to CXCR7, the experiments were also conducted in MCF-7, human breast cancer cell line, overexpressing CXCR7, using anti-CXCR7 APC-antibody (FAB4227A clone 11G8 R&D Systems, Minneapolis, MN, USA). Briefly, 5 × 10^5^ MCF-7cells were pre-incubated with anti-CXCR7 (clone 11G8) (10ug/ml), CXCL11 (100ng/ml), CXCL12 (100ng/ml) and CCX771 (100nM) in the binding buffer (PBS 1x plus 0.2% BSA and 0.1% NaN3) for 45 minutes at 37° C, 5% CO_2_ and then labeled for 30 minutes using anti-CXCR7 APC-antibody. The cells were counted through a FACS Canto II cytofluorometer (Becton Dickinson Immunocytometry Systems, Mountain View, CA, USA).

*Migration*. Migration was assayed in 24-well Transwell chambers (Corning Inc., Corning, NY) using inserts 8-µm pore membranes. Membranes were pre-coated with collagen (human collagen type I/III) and fibronectin (20 µg/mL each). Cells were placed in the upper chamber (2 × 10^5^cells/well) in culture medium containing 0.5% BSA (migration media) in the presence of the anti-CXCR7 (11G8, R&D System) (10µg/ml) or CCX771, a small molecule inhibitor of CXCR7; 100 ng/mL CXCL12 was added to the lower chamber. After 18 h incubation, the cells were fixed in 4% (w/v) paraformaldehyde in PBS and stained for 15 minutes with DAPI. Cells that had migrated to the bottom side of the membrane were visualized under the fluorescent microscope (Carlo Zeiss, Axio Scope.A1) and counted (cells in five randomly chosen visual fields). The migration index was defined as the ratio between migrating cells in the experimental group and migrated cells in the control group.
